# Molecular imaging of atherosclerotic plaque vulnerability: a narrative review

**DOI:** 10.1007/s10554-026-03724-7

**Published:** 2026-05-14

**Authors:** Victoria To, Jason Ha

**Affiliations:** 1 GKT School of Medical Education, King’s College London, London, UK; 2 Bristol Medical School, University of Bristol, Bristol, UK

**Keywords:** Atherosclerosis, Molecular imaging, Plaque vulnerability, Inflammation, Microcalcification, Angiogenesis

## Abstract

Atherosclerotic plaque rupture or erosion underlies most acute coronary and cerebrovascular events. Risk stratification currently relies on luminal stenosis, which poorly predicts thrombotic risk because many events arise from biologically vulnerable plaques with only mild-to-moderate narrowing. Molecular imaging enables non-invasive assessment of the biological processes that drive plaque instability. A structured narrative review of experimental and clinical studies published between 2000 and 2024 was conducted using PubMed and Ovid Embase. Searches related to atherosclerosis, vulnerable plaque, molecular imaging, inflammation, microcalcification, matrix remodelling, angiogenesis, and carotid or coronary disease identified 2,627 records, of which 38 studies were included after screening. Imaging of inflammation and microcalcification shows the greatest clinical maturity, primarily using positron emission tomography (PET). Inflammation is assessed with ¹⁸F-fluorodeoxyglucose (¹⁸F-FDG), reflecting macrophage metabolic activity, while active microcalcification is detected with ¹⁸F-sodium fluoride (¹⁸F-NaF), which binds to hydroxyapatite in early calcific deposits. Imaging of fibrous cap degeneration and angiogenesis remains less clinically mature with limited human validation, including matrix metalloproteinase-targeted single-photon emission computed tomography (SPECT) and integrin-targeted magnetic resonance imaging (MRI) approaches. Translation is limited by technical complexity, cost, and scarce outcome-based validation. Molecular imaging provides biologically informed assessment of plaque vulnerability beyond stenosis alone. Imaging of inflammation and microcalcification currently show the greatest potential for risk stratification, although further validation and multimodal integration are required before routine clinical use.

## Introduction

Cardiovascular disease (CVD) remains a leading cause of mortality worldwide, with atherosclerosis underlying most acute ischaemic coronary and cerebrovascular events. These events most commonly arise from thrombosis triggered by plaque rupture or endothelial erosion. Accurate prediction of plaque destabilisation risk is therefore central to prevention of thrombotic events.

Current risk stratification for atherosclerosis remains primarily focused on imaging-based anatomical assessment of luminal stenosis [1]. This metric underpins eligibility for surgical and interventional management. Imaging techniques such as Doppler ultrasound and computed tomography angiography are widely used to quantify vessel narrowing [2]. Whilst these modalities provide high-resolution structural information, they offer limited insight into plaque biological activity and composition. These limitations are clinically significant, as stenosis severity alone is an unreliable predictor of thrombotic risk: most acute coronary and carotid ischaemic events arise from lesions causing < 50% and < 70% luminal obstruction, respectively [3]. This is largely because thrombotic risk is governed by a complex interplay of biological processes beyond stenosis, including inflammation, structural instability, and adverse remodelling.

Molecular imaging has consequently continued to gain interest as an adjunctive approach in the assessment of atherosclerosis. In contrast to conventional anatomical imaging, molecular imaging enables visualisation and quantification of biologically active processes within plaques that drive plaque destabilisation and vulnerability. By characterising plaque biology rather than luminal narrowing alone, molecular imaging has the potential to improve identification of high-risk lesions and refine prediction of future thrombotic events. However, imaging of individual plaque characteristics in isolation has not consistently outperformed measures of overall atherosclerotic disease burden in predicting clinical events. This suggests that the clinical utility of molecular imaging lies in multimodal approaches that combine complementary techniques to capture different biological pathways across the arterial tree and enable longitudinal assessment of disease activity [4]. By integrating information across multiple vascular territories and biological processes, such approaches more closely reflect the systemic nature of atherosclerosis. This aligns with the vulnerable patient framework, which posits that thrombotic risk is driven by systemic and disease-wide processes rather than individual plaques alone. Despite these advances, the clinical role of molecular imaging in risk stratification of atherosclerosis remains incompletely defined, with heterogeneous evidence across imaging modalities and biological targets and significant barriers to routine implementation.

This narrative review synthesises experimental and clinical evidence on molecular imaging strategies targeting inflammation, micro calcification, fibrous cap degeneration, and angiogenesis, with a primary focus on carotid and coronary atherosclerotic plaques. It evaluates the translational potential and current limitations of these techniques in improving clinical risk stratification beyond stenosis-based assessment.

## Methods

### Study design

This narrative review was conducted to synthesise contemporary evidence on molecular imaging approaches for assessing atherosclerotic plaque vulnerability. Studies were selected to represent key biological targets, imaging modalities, and clinically relevant themes, with emphasis on methodological rigour, biological validation, and translational relevance.

### Search strategy

Searches were conducted for studies published between January 2000 and December 2024 using PubMed and OVID Embase. Search terms were structured around atherosclerosis, plaque vulnerability, molecular imaging modalities, and key biological processes associated with plaque instability. Representative terms included “atherosclerosis”, “vulnerable plaque”, “molecular imaging”, “positron emission tomography (PET)”, “magnetic resonance imaging (MRI)”, “FDG PET”, “NaF PET”, “vascular inflammation”, “microcalcification”, “matrix metalloproteinase”, and “angiogenesis”, along with artery-specific terms such as “carotid” and “coronary” atherosclerosis.

### Screening

The search yielded 2627 records. After removing duplicates, titles and abstracts of 1824 papers were screened by two independent reviewers for relevance to the review objectives. We favoured articles that presented detailed characterisation of imaging methodology, biological targets, and clinical or histological validation where available. Articles considered potentially relevant were selected for full-text review. Additional studies, including seminal publications, were identified through manual review of reference lists and citation tracking. A total of 38 studies were included in the final synthesis.

### Data extraction

The included studies were analysed for information relating to imaging modality, biological target, study design, study population (human or preclinical), vascular territory examined, and key findings pertaining to plaque vulnerability. Given the narrative design of this review, data extraction was performed to support qualitative synthesis rather than quantitative pooling. We prioritised relevant texts to highlight the four major biological themes, rather than presenting an exhaustive overview of the entire body of literature with strict inclusion and exclusion criteria. By focusing on qualitative interpretation and synthesis of the literature, our narrative review differs from, but complements, a quantitative approach.

## Results

### Overview

**Fig. 1:**
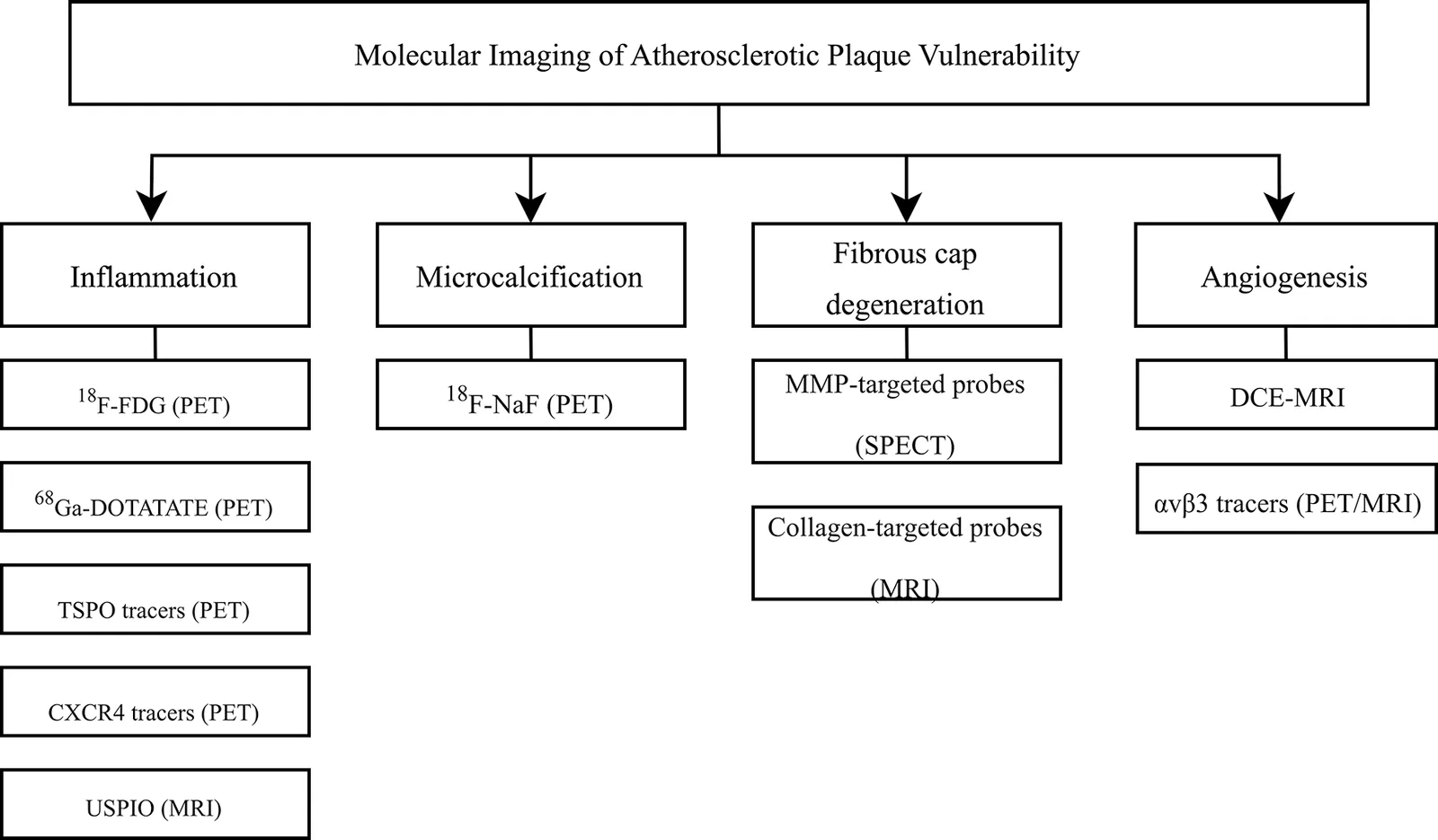
Overview of molecular imaging approaches targeting key biological determinants of atherosclerotic plaque vulnerability

A total of 38 studies were included in the final synthesis, spanning four major biological determinants of atherosclerotic plaque vulnerability: inflammation, microcalcification, fibrous cap degeneration, and angiogenesis. Included studies ranged from preclinical animal models to human imaging and histological validation studies, encompassing a variety of molecular imaging modalities, including PET, SPECT, and MRI. The findings are presented thematically across these four biological targets, with each section addressing the underlying pathobiology, followed by the available molecular imaging approaches and their translational limitations. The key biological processes underlying plaque vulnerability and corresponding molecular imaging approaches are summarised in Fig. 1. The characteristics of the included studies are summarised in Table 1.

**Table 1:** Study characteristics of included studies

Author	Year	Country	Biological theme	Main findings
Trivedi et al.	2006	UK	Inflammation	USPIO-enhanced MRI identifies macrophage-rich inflamed carotid plaques in vivo.
Samaržija et al.	2022	Croatia	None (structural stenosis)	CTA cross-sectional area measurements correlated better with Doppler ultrasound than diameter-based methods for grading carotid stenosis.
Fayad et al.	2024	USA	None	Overall atherosclerotic disease burden predicts cardiovascular events more reliably than individual plaque characteristics.
Achenbach	2023	Germany	Fibrous cap degeneration	Plaque rupture is multifactorial; vulnerable plaques may be only mildly stenotic.
Flynn et al.	2019	Australia	Inflammation	Monocytes/macrophages drive atherosclerotic progression and instability.
Libby et al.	2013	USA	Inflammation	Monocyte subsets have distinct roles in plaque inflammation and progression.
Tawakol et al.	2006	USA	Inflammation	¹⁸F-FDG PET uptake in carotid plaques correlates strongly with macrophage density on histology.
Rudd et al.	2008	UK	Inflammation	¹⁸F-FDG PET signal shows high short-term reproducibility, supporting its use for monitoring plaque inflammation.
Masteling et al.	2011	Netherlands	Inflammation	High-resolution FDG microPET demonstrates heterogeneous uptake within plaques that correlates with macrophage infiltration.
Dweck et al.	2016	UK	Multiple	Reviews molecular imaging of atherosclerosis, highlighting FDG-PET (inflammation) and NaF-PET (microcalcification) as key tools for identifying high-risk patients.
Tarkin et al.	2017	UK	Inflammation	⁶⁸Ga-DOTATATE PET identifies macrophage-driven plaque inflammation and outperforms FDG for detecting high-risk coronary lesions.
Hellberg et al.	2017	Finland	Inflammation	¹⁸F-FEMPA PET targets TSPO-expressing macrophages and shows uptake in atherosclerotic plaques that correlates with macrophage density.
Lu et al.	2022	Austria	Inflammation	⁶⁸Ga-PentixaFor PET/MRI detects CXCR4-mediated vascular inflammation and identifies more atherosclerotic lesions with higher uptake than FDG.
Lu et al.	2021	USA	Inflammation	Ferumoxytol-based MRI visualises macrophage-driven inflammation in cardiovascular disease.
Tang et al.	2009	UK	Inflammation	USPIO-enhanced MRI detects macrophage infiltration within atherosclerotic plaques, indicating inflammatory activity.
Joshi et al.	2014	UK	Microcalcification	18 F-NaF PET helps identify vulnerable plaques via microcalcification activity.
Wen et al.	2022	China	Microcalcification	Coronary ¹⁸F-NaF PET uptake correlated with histological markers of plaque vulnerability, and showed good discriminatory ability for identifying high-risk plaques.
Nakahara et al.	2017	USA	Microcalcification	Microcalcification reflects active plaque instability and can be detected by ¹⁸F-NaF PET, whereas large calcifications are generally associated with stability.
Puchner et al.	2018	USA	Microcalcification	Total coronary calcium burden predicts ACS risk, whereas dense local calcification is associated with plaque stability rather than culprit lesions.
Kwiecinski et al.	2020	UK/USA	Microcalcification	¹⁸F-NaF PET predicts future myocardial infarction in patients with coronary artery disease, with increased uptake associated with > 7-fold higher risk.
Tzolos and Dweck	2020	UK	Microcalcification	¹⁸F-NaF PET detects active microcalcification and vascular calcification activity that is not visible on conventional CT.
Alonso-Herranz et al.	2023	Denmark/Spain	Fibrous cap degeneration	Fibrous caps are formed by smooth muscle cell–derived collagen layers, and cap thinning or rupture is the primary trigger of thrombosis.
Hatsukami et al.	2000	USA	Fibrous cap degeneration	High-resolution MRI can identify fibrous cap thickness and rupture in vivo, distinguishing stable from high-risk plaques.
Schäfers et al.	2004	Germany	Fibrous cap degeneration	Radiolabelled MMP inhibitors allow in-vivo scintigraphic imaging of MMP activity, implicated in extracellular matrix breakdown and plaque rupture risk.
Hakimzadeh	2017	Iran/Europe	Fibrous cap degeneration	Novel radiolabelled MMP-2/9 inhibitors enable imaging of extracellular matrix degradation processes associated with plaque instability and rupture.
Chen et al.	2013	USA/Spain	Fibrous cap degeneration	Collagen-targeted HDL nanoparticles (EP3533-HDL) enable MRI detection of plaque regression, with increased signal corresponding to rising collagen content and reduced macrophage burden.
Kawai et al.	2024	Netherlands	Fibrous cap degeneration	Unstable plaques are characterised by thin fibrous caps, large necrotic cores, macrophage infiltration, and thrombosis.
Calcagno et al.	2010	USA	Angiogenesis	DCE-MRI can non-invasively quantify plaque neovascularisation, a key feature of vulnerable plaques linked to inflammation.
Truijman et al.	2013	Netherlands	Inflammation + Angiogenesis	FDG-PET and DCE-MRI showed a weak but significant correlation between plaque inflammation and neovascularisation.
Van Hoof et al.	2017	Netherlands	Angiogenesis	Dynamic contrast-enhanced MRI (DCE-MRI) parameters of the vessel wall and adventitia correlated with markers of plaque microvascular permeability.
Botnar et al.	2015	Germany	Multiple	Molecular MRI with targeted probes can visualise plaque biology, including macrophages, angiogenesis, and extracellular matrix changes.
Winter et al.	2003	USA	Angiogenesis	αvβ3-integrin–targeted nanoparticles enabled MRI detection of plaque neovascularisation in early atherosclerosis, reflecting angiogenic activity.
Jenkins et al.	2014	UK	Angiogenesis	¹⁸F-fluciclatide PET detects αvβ3 integrin expression in human atherosclerosis, correlating with plaque burden.
Tawakol et al.	2013	USA	Inflammation	High-dose atorvastatin significantly reduced arterial ¹⁸F-FDG uptake (target-to-background ratio) within 12 weeks.
Mateo et al.	2014	Spain/USA	Hypoxia	¹⁸F-FMISO PET detects plaque hypoxia, which increases with disease progression and co-localises with macrophage-rich, angiogenic regions.
Laufer et al.	2008	Netherlands/USA	Apoptosis	Annexin A5 imaging detects apoptosis in atherosclerotic plaques, with uptake correlating with macrophage-rich, unstable lesions.
Nadel et al.	2024	UK	Oxidative stress	MPO-targeted MRI detects myeloperoxidase activity in atherosclerotic plaques and identifies inflamed, rupture-prone lesions.
Boutagy et al.	2018	USA	Oxidative stress	¹⁸F-DHMT PET detects early increases in reactive oxygen species, preceding structural damage.

### Inflammation

Atherosclerosis is a chronic inflammatory disease initiated by endothelial injury, which permits subendothelial accumulation and oxidation of low-density lipoprotein. Oxidised LDL promotes macrophage recruitment and foam cell formation within the arterial intima, initiating a self-perpetuating inflammatory cascade that drives fatty streak formation [5]. Progression from fatty streaks to vulnerable atherosclerotic plaques is driven by an imbalance between pro-inflammatory and reparative immune processes within the plaque, with macrophages acting as central regulators of this balance. Plaque macrophages exist along a continuous phenotypic spectrum shaped by the local microenvironment, with pro-inflammatory subsets promoting defective efferocytosis, necrotic core expansion, and fibrous cap thinning, whereas reparative phenotypes support matrix deposition and plaque stabilisation [6]. Predominance of pro-inflammatory signalling therefore promotes accumulation of uncleared apoptotic macrophages and expansion of a thrombogenic necrotic core, a defining feature of vulnerable plaques and a key driver of acute atherothrombotic events. Whilst macrophage phenotypes exist along a spectrum of inflammatory states, increased macrophage burden and activity is consistently associated with heightened plaque inflammation. Macrophages therefore represent an attractive biological target for imaging plaque inflammation and, by extension, plaque vulnerability. ¹⁸F-fluorodeoxyglucose positron emission tomography (¹⁸F-FDG PET) has consequently been utilised to assess vascular inflammation indirectly, with uptake quantified via target-to-background ratio [7].

As a glucose analogue, ¹⁸F-FDG is preferentially taken up by metabolically active macrophages via GLUT transporters. Thus, vascular tracer uptake reflects glycolytic activity and serves as a proxy for inflammatory burden. The validity of ¹⁸F-FDG PET as a surrogate marker of plaque inflammation has been supported by histological correlation in human studies. Rudd et al. (*r* = 0.80, *p* < 0.001) and Masteling et al. (*r* = 0.68, *p* < 0.01) demonstrated strong positive associations between FDG uptake and CD68 + macrophage density in excised carotid plaques, providing direct evidence that increased tracer uptake reflects inflammatory cell burden [8, 9]. Hybrid PET/CT or PET/MR imaging further enables anatomical localisation of inflammatory signals within the vessel wall, facilitating differentiation between plaque and adjacent tissues [10]. In practice, ¹⁸F-FDG PET has been most extensively applied in large-vessel atherosclerosis, particularly in the carotid arteries and aorta, whereas coronary imaging remains technically challenging due to physiological myocardial FDG uptake and limited spatial resolution. Beyond ¹⁸F-FDG, several alternative PET tracers have been developed to improve specificity for inflammatory cell subtypes and overcome limitations related to non-specific myocardial uptake. Somatostatin receptor imaging using ⁶⁸Ga-DOTATATE targets somatostatin receptor subtype-2 expressed on activated macrophages and has demonstrated improved signal-to-background ratios compared with FDG, particularly in coronary imaging [11]. Translocator protein (TSPO)–targeted tracers have also been investigated as markers of inflammatory cell activation, including macrophages, reflecting mitochondrial activity within these cells. However, their clinical application is limited by inter-individual variability in binding affinity [12]. More recently, CXCR4-targeted imaging has emerged as a promising approach to assess leukocyte recruitment and inflammatory cell trafficking within atherosclerotic plaques, providing complementary insight into inflammatory signalling pathways [13]. These approaches may offer greater biological specificity than FDG, although their clinical role remains under investigation.

Contrast-enhanced MRI has also been employed to assess macrophage-driven inflammation within atherosclerotic plaques. Ultrasmall superparamagnetic iron oxide (USPIO) particles act as macrophage-targeted contrast agents and are phagocytosed following intravenous administration [1]. After cellular uptake, USPIOs such as ferumoxytol create local magnetic field inhomogeneities, leading to T2 signal shortening and the appearance of focal hypointense regions on T2-weighted MRI [1]. The extent and intensity of these signal changes can be quantified, providing a surrogate marker of macrophage burden within the plaque. Human imaging studies have demonstrated preferential USPIO accumulation within vulnerable atherosclerotic lesions. Lu et al. reported USPIO uptake across 75% of the surface area of histologically defined vulnerable carotid plaques, compared with only 7% in stable lesions, supporting the use of USPIO-enhanced MRI as a quantitative marker of plaque macrophage burden [14]. In addition, Tang et al. demonstrated a significant reduction in carotid plaque USPIO uptake following three months of high-dose statin therapy, highlighting the potential of USPIO-enhanced MRI for monitoring therapeutic modulation of vascular inflammation [15]. To date, USPIO-enhanced MRI has been evaluated predominantly in the carotid arteries. Compared with ¹⁸F-FDG PET, it offers higher spatial resolution and avoids contamination from physiological myocardial FDG uptake, a recognised limitation of FDG-based coronary imaging. However, clinical translation of USPIO-enhanced MRI remains constrained by limited availability of approved contrast agents, delayed imaging protocols, and technical challenges in signal interpretation.

Importantly, these imaging modalities capture distinct but complementary aspects of plaque inflammation. ¹⁸F-FDG PET reflects glycolytic activity in metabolically active macrophages, USPIO-enhanced MRI provides information on macrophage phagocytic burden, and newer tracers such as ⁶⁸Ga-DOTATATE, TSPO-targeted agents, and CXCR4-based imaging offer additional insight into macrophage activation and leukocyte recruitment. Together, these approaches may improve biological characterisation of plaque inflammation and support more refined risk stratification. However, although multimodal imaging is conceptually attractive, evidence that combining modalities improves prediction beyond single-tracer imaging remains limited, and further studies are needed to define its clinical utility.

### Microcalcification

Microcalcification is a key determinant of atherosclerotic plaque stability, with distinct biological and mechanical implications depending on its morphology. Early-stage microcalcifications arise in inflamed plaques and are characterised by small, dispersed deposits that act as focal points which concentrate mechanical stress within the fibrous cap, increasing plaque vulnerability [16]. In contrast, late-stage macrocalcifications form larger, confluent deposits that distribute stress more evenly and are generally associated with plaque stabilisation [17]. Microcalcification is increasingly recognised as a downstream consequence of plaque inflammation, arising from apoptotic macrophages and matrix vesicles within the necrotic core [18]. This mechanistic link suggests that ¹⁸F-NaF uptake may capture a later stage of the same inflammatory cascade detected by ¹⁸F-FDG PET [18].

Multislice computed tomography (MSCT) is well established for the detection of macrocalcifications due to the high radiodensity of calcium deposits [19]. In clinical practice, coronary artery calcium (CAC) scoring quantifies the total burden of calcified coronary atherosclerosis. Although dense macrocalcification may indicate individual plaque stability, higher CAC scores reflect overall disease burden and future cardiovascular risk [17]. However, CAC scoring lacks sensitivity to early microcalcifications and provides limited insight into the vulnerability of individual plaques.

In contrast, molecular imaging approaches enable in vivo assessment of microcalcification. ¹⁸F-sodium fluoride (¹⁸F-NaF) has emerged as a leading PET tracer for imaging early-stage microcalcification. The tracer binds to exposed hydroxyapatite on the surface of nascent calcific deposits, forming fluorapatite and generating a detectable PET signal [20]. Consequently, tracer uptake preferentially occurs in microcalcifications, where hydroxyapatite remains accessible, whereas uptake is reduced in advanced macrocalcified plaques, in which hydroxyapatite becomes internalised [20]. Histological validation studies in carotid atherosclerosis have demonstrated that focal ¹⁸F-NaF uptake co-localises with areas of active microcalcification on microscopy, while densely macrocalcified regions exhibit comparatively low tracer uptake [17]. In contrast, although ¹⁸F-NaF PET has been applied to coronary atherosclerosis, direct histological validation of tracer uptake remains limited. Instead, coronary evidence is derived predominantly from in vivo imaging studies in which increased ¹⁸F-NaF uptake localises to culprit plaques following myocardial infarction, representing event-based validation rather than direct histological confirmation of microcalcification [19]. Beyond culprit lesion identification, prospective studies have demonstrated that elevated coronary ¹⁸F-NaF uptake independently predicts future myocardial infarction, with risk estimates exceeding those of conventional markers including CAC scoring [21]. This positions ¹⁸F-NaF PET as a potential tool for identifying high-risk individuals prior to clinical events. The integration of PET with CT enables anatomical localisation of tracer signal and discrimination between biologically active microcalcification and established, dormant macrocalcification. Despite this progress, routine clinical adoption of ¹⁸F-NaF PET–CT remains limited by cost, radiation exposure, and the absence of established management algorithms for incidentally detected high-risk plaques. Demonstrating that ¹⁸F-NaF–guided intervention improves outcomes over standard care will be essential for clinical integration.

### Fibrous cap degeneration

The fibrous cap plays a critical role in maintaining plaque stability by separating the thrombogenic necrotic core from the arterial lumen [22]. Plaque vulnerability is influenced by its structural integrity, determined by a balance between extracellular matrix synthesis by vascular smooth muscle cells (VSMCs) and matrix degradation by proteolytic enzymes such as matrix metalloproteinases (MMPs) [22]. Excessive matrix degradation weakens the fibrous cap, increasing susceptibility to rupture or erosion and subsequent thrombus formation. Importantly, fibrous cap degradation represents a downstream manifestation of inflammatory and proteolytic activity within the plaque, suggesting that imaging matrix degradation may provide complementary information to inflammatory and calcific imaging approaches.

Although high-resolution MRI can assess fibrous cap thickness in vivo, predominantly in carotid atherosclerosis, it provides structural rather than molecular information and therefore cannot directly capture biological processes driving cap weakening [23]. To address this limitation, molecular imaging approaches, most notably matrix metalloproteinase (MMP)–targeted single-photon emission computed tomography (SPECT), have been investigated to assess proteolytic activity associated with fibrous cap degeneration. MMP activity therefore serves as a surrogate marker of extracellular matrix degradation within the atherosclerotic plaque. In preclinical studies, de novo radioligands with high affinity for multiple MMP subtypes have demonstrated focally increased tracer uptake at sites of vascular injury in ApoE-deficient mice compared with wild-type controls [24]. More recently, Hakimzadeh et al. developed a radioligand specifically targeting MMP-2 and MMP-9, gelatinases implicated in degradation of type I and type IV collagen and elastin fibres within the fibrous cap [25]. However, matrix metalloproteinase activity is not specific to fibrous cap rupture and may also reflect broader vascular remodelling and inflammatory processes, limiting the specificity of MMP-targeted imaging as a direct marker of plaque rupture risk. Current approaches therefore primarily enable detection of proteolytic activity rather than robust quantification of fibrous cap degeneration. Evidence also remains confined to animal models without established human validation. Consequently, while MMP-targeted molecular imaging provides biologically relevant insight into processes associated with fibrous cap weakening, its clinical utility in risk stratification remains limited at present.

In addition to imaging matrix degradation, collagen-targeted imaging, primarily using MRI based contrast agents, has emerged as a complementary approach for assessing fibrous cap integrity. Preclinical studies using collagen-binding probes, including CNA35-based tracers and synthetic collagen-binding peptides, demonstrate preferential uptake in regions of preserved fibrous structure, suggesting potential utility in distinguishing stable from high-risk plaques [26]. However, evidence remains limited to preclinical and early translational studies, and clinical application has yet to be established.

### Angiogenesis

Angiogenesis refers to the formation of neovessels within and surrounding atherosclerotic plaques, a process driven primarily by hypoxia as plaque growth exceeds the diffusion capacity of oxygen from the arterial lumen [27]. These neovessels are structurally immature and highly permeable, facilitating inflammatory cell infiltration and lipoprotein deposition. Their fragility also predisposes plaques to intraplaque haemorrhage, whereby extravasated erythrocytes contribute membrane-derived cholesterol and haemoglobin-associated iron to the necrotic core, accelerating necrotic core expansion and promoting plaque instability [28]. Through these mechanisms, angiogenesis establishes a pathological feedback loop with inflammation and necrotic core progression, as inflammatory signalling and intraplaque haemorrhage further stimulate neovascularisation and amplify plaque vulnerability.

Dynamic contrast-enhanced MRI (DCE-MRI) enables assessment of plaque neovascularisation. Following intravenous administration of a gadolinium-based contrast agent, serial T1-weighted imaging allows estimation of the volume transfer constant (Ktrans) [28]. Ktrans reflects contrast leakage from the plasma into the extravascular space and therefore serves as a surrogate marker of plaque microvascular permeability rather than directly measuring microvessel density. In human studies, DCE-MRI has been applied predominantly in carotid atherosclerosis. Truijman et al. demonstrated lower Ktrans values within the lipid-rich necrotic core compared with the highly vascularised outer plaque regions in carotid plaques, consistent with increased neovascular density at the plaque periphery and adventitia [29]. Histological validation studies have further demonstrated moderately strong correlations between Ktrans and CD31-positive microvessel density in carotid endarterectomy specimens (Pearson’s ρ = 0.59–0.72) [30]. Consequently, although Ktrans does not directly quantify vessel number, it provides a validated functional imaging biomarker of plaque microvascular permeability and angiogenic activity.

Beyond functional assessment, MRI can also be adapted for molecular imaging of angiogenesis using targeted contrast agents. Whereas DCE-MRI captures the functional consequences of neovascularisation through increased vascular permeability, integrin-targeted imaging enables more direct visualisation of the molecular drivers of angiogenesis. Angiogenic biomarkers such as the αvβ3 integrin are upregulated during neovessel formation and facilitate expansion of the vasa vasorum into the plaque [31]. In a preclinical study, Winter et al. demonstrated in vivo localisation of αvβ3 expression using gadolinium-based integrin-targeted contrast agents, showing increased T1 signal intensity at sites of angiogenesis with strong concordance with histological measures of neovascular density [32]. Complementing MRI-based approaches, αvβ3 integrin–targeted PET tracers have also been developed to enable sensitive detection of angiogenic activity. These tracers, including ¹⁸F-flotegatide, bind to αvβ3 integrins expressed on activated endothelial cells and inflammatory cells such as macrophages, allowing in vivo assessment of processes related to both angiogenesis and inflammation within the plaque [33]. In contrast to MRI-based techniques, PET imaging offers greater sensitivity and enables quantitative assessment of tracer uptake, facilitating detection of low-level angiogenic activity across the vascular tree. Early preclinical studies have demonstrated that αvβ3-targeted PET imaging can localise areas of active neovessel formation, although clinical application remains limited and requires further validation.

Despite these promising findings, translation of targeted MRI and PET approaches for angiogenesis remains largely confined to preclinical models, reflecting ongoing challenges related to contrast agent safety, sensitivity, and regulatory approval. Should these barriers be overcome, integrin-targeted imaging could provide a non-invasive means of identifying plaques at the earliest stages of pathological neovascularisation, before the development of intraplaque haemorrhage or fibrous cap thinning.

## Discussion

### Comparison of imaging targets

The integration of molecular imaging modalities has the potential to extend atherosclerosis assessment beyond luminal stenosis and towards biological characterisation of plaque vulnerability. Across the major biological determinants of plaque instability, the degree of clinical translation of available imaging techniques varies considerably. Inflammatory activity and microcalcification are the most clinically advanced targets, with PET-based imaging using established tracers (e.g. ¹⁸F-FDG, ¹⁸F-NaF) demonstrating validation in human studies across both carotid and coronary vascular beds. Both tracers have progressed beyond histological validation to demonstrate clinical utility: ¹⁸F-FDG PET has been employed as a surrogate endpoint in interventional trials assessing anti-inflammatory therapies [34], while prospective studies have shown that elevated coronary ¹⁸F-NaF uptake is associated with increased risk of subsequent myocardial infarction [21], outperforming conventional risk predictors including coronary artery calcium scoring. In contrast, imaging of fibrous cap degeneration and angiogenesis remains at an earlier stage of clinical translation. Fibrous cap degeneration imaging, most commonly through matrix metalloproteinase–targeted SPECT, remains confined to preclinical animal models, with no robust human validation to date. Similarly, angiogenesis imaging has limited clinical integration. Functional assessment using DCE-MRI has been validated only in carotid atherosclerosis, while molecularly targeted approaches, including αvβ3 integrin imaging, remains limited, with most molecular approaches confined to preclinical models and only early human validation available. Consequently, imaging of fibrous cap degeneration and angiogenesis remains further from routine clinical translation than inflammatory and calcific imaging, reflecting both limited human validation and substantial technical and regulatory challenges.

### Additional imaging targets

Although this comparison highlights key clinically relevant imaging targets, this narrative review is not exhaustive and does not cover all emerging pathways in atherosclerosis. Hypoxia imaging is one example. As plaques enlarge, oxygen diffusion becomes more limited and the plaque core may become hypoxic, making hypoxia imaging a surrogate marker of metabolic stress, angiogenesis, and plaque progression. Nitroimidazole tracers such as ^18^F-FMISO diffuse into cells and are enzymatically reduced under low-oxygen conditions. In normoxic tissue, they are reoxidised and washed out, whereas in hypoxic regions they remain trapped intracellularly, allowing PET-based quantification of hypoxic burden [35]. Moreover, apoptosis and necrosis occur in atherosclerotic plaques due to oxidative stress and impaired clearance of dying cells, contributing to cell debris accumulation and necrotic core expansion and thereby promoting plaque instability. Annexin V–based tracers detect apoptosis by binding to phosphatidylserine exposed on the outer membrane during early apoptosis, enabling non-invasive quantification of apoptotic activity within plaques [36]. Oxidative stress imaging provides a complementary approach by targeting the upstream redox injury that drives these cell death pathways. MRI-based myeloperoxidase-targeted probes have been used to detect myeloperoxidase activity, an enzyme released by inflammatory cells and implicated in oxidative injury within plaques [37]. Emerging PET tracers, including ^18^F-DHMT, are being developed to detect reactive oxygen species or related oxidative products in situ [38]. While validated in other inflammatory models, their application to atherosclerotic plaque imaging remains investigational. Among these pathways, hypoxia imaging appears the most clinically advanced, given human PET studies with nitroimidazole tracers in carotid and coronary atherosclerosis. Apoptosis imaging shows promising early human validation, while oxidative stress imaging remains predominantly preclinical, despite growing interest in myeloperoxidase-targeted MRI and ROS-sensitive PET tracers.

### Translational barriers and future directions

Taken together, these findings reflect broader barriers that continue to limit the routine clinical implementation of molecular imaging approaches, even for the more established tracers. Large-scale prospective studies demonstrating incremental value over conventional anatomical imaging remain limited, and any clinical benefit must be substantial to justify the additional radiation exposure, cost, and logistical complexity associated with molecular imaging. Technical challenges also persist, including motion artefact in coronary imaging, physiological myocardial FDG uptake that limits coronary ¹⁸F-FDG PET assessment, and the requirement for delayed imaging protocols with certain contrast agents. Furthermore, regulatory approval of novel radiotracers and targeted contrast agents remains a major hurdle, particularly for molecularly targeted approaches such as integrin-targeted MRI and MMP-targeted SPECT. Bridging this translational gap will require development of safer molecular probes, standardised imaging protocols amenable to multicentre validation, and ultimately, prospective evidence demonstrating that molecular imaging can meaningfully influence clinical decision-making and improve patient outcomes.

## Conclusion

Molecular imaging represents a powerful approach for advancing the assessment of atherosclerotic plaque vulnerability. However, key translational challenges, including technical complexity, tracer safety, and the need for robust human validation, must be addressed to enable wider clinical adoption. Once these barriers are overcome, the development of standardised frameworks integrating molecular imaging signals across multiple biological processes may enable robust risk stratification based on plaque biology, beyond luminal stenosis alone. This integrative approach has the potential to support personalised preventive and interventional strategies based on individual plaque biology, representing a clinically meaningful direction for the future management of atherosclerotic disease.

## Data Availability

No datasets were generated or analysed during the current study.
